# THE ASSOCIATIONS BETWEEN PERSONAL EVENTS AND COGNITIVE FUNCTION: DOES GENDER MAKE A DIFFERENCE?

**DOI:** 10.1093/geroni/igz038.3539

**Published:** 2019-11-08

**Authors:** Yingxiao Hua, Gabriella Dong

**Affiliations:** Rutgers, The State University of New Jersey, New Brunswick, New Jersey, United States

## Abstract

Recent studies suggested stressful personal events were associated with lower cognitive function and that men and women have different responses to stressful events. There is additionally ample evidence supported gender difference in cognitive function in later age. However, research regarding whether gender would moderate the relationship between personal events and cognitive function are limited. Our data were retrieved from 3,126 US Chinese older adults in Chicago between 2017 to 2019. Personal events were measured by a composite score (range 0-28) of all lifetime events including fire, physical assault, rob, sexual assault, divorce, death, cancer, homeless, imprisonment, false accuse, miscarriage and abortion. Global cognition was constructed by mean of z scores from five cognition tests, covering episodic memory, working memory, executive function and C-MMSE test. Stepwise linear regressions were performed to test the association. After adjusting for social demographic confounders, personal events score was positively associated with global cognition (b=0.018, SE=0.007, p=0.013), and significant interaction term of gender and global cognition was found (b=0.019, SE=0.009, p=0.041). The study found the protective effect of undergoing personal events on cognitive function in an aging population, and it was stronger among women. The findings highlighted the important role of gender in the relationship. Identifying mechanisms underlying this may provide gender-specific information for prevention of cognition decline among older adults. Future studies could explore gender difference in short-term and chronic stressful personal events, respectively, to improve understandings of how traumatic personal events impact cognitive function.

